# Effectiveness of Core Stability Exercises and Recovery Myofascial Release Massage on Fatigue in Breast Cancer Survivors: A Randomized Controlled Clinical Trial

**DOI:** 10.1155/2012/620619

**Published:** 2011-07-17

**Authors:** Irene Cantarero-Villanueva, Carolina Fernández-Lao, Rosario del Moral-Avila, César Fernández-de-las-Peñas, María Belén Feriche-Fernández-Castanys, Manuel Arroyo-Morales

**Affiliations:** ^1^Department of Physical Therapy, Health Sciences Faculty, University of Granada, 18071 Granada, Spain; ^2^Radiotherapy Breast Oncology Unit, University Hospital Virgen de las Nieves, Granada, Spain; ^3^Department of Physical Therapy, Occupational Therapy, Rehabilitation, and Physical Medicine, King Juan Carlos University, Alcorcón, Spain; ^4^Physical Education Department, Faculty of Sciences, Physical Activity, and Sports, University of Granada, 18071 Granada, Spain

## Abstract

The purpose of the present paper was to evaluate the effects of an 8-week multimodal program focused on core stability exercises and recovery massage with DVD support for a 6-month period in physical and psychological outcomes in breast cancer survivors. A randomized controlled clinical trial was performed. Seventy-eight (*n* = 78) breast cancer survivors were assigned to experimental (core stability exercises plus massage-myofascial release) and control (usual health care) groups. The intervention period was 8 weeks. Mood state, fatigue, trunk curl endurance, and leg strength were determined at baseline, after the last treatment session, and at 6 months of followup. Immediately after treatment and at 6 months, fatigue, mood state, trunk curl endurance, and leg strength exhibited greater improvement within the experimental group compared to placebo group. This paper showed that a multimodal program focused on core stability exercises and massage reduced fatigue, tension, depression, and improved vigor and muscle strength after intervention and 6 months after discharge.

## 1. Introduction

Almost all breast cancer survivors (BCS) suffer from one or more cancer-related symptoms that impact their quality of life. Multimodal therapeutic programs can ameliorate and reduce the patient's impairments by improving their ability to carry out daily tasks [1]. Nevertheless, health care practitioners feel that their practice is usually affected by the lack of exercise guidance for cancer population suffering from fatigue-related cancer [2].

 One principal component of a multimodal program is the therapeutic exercise. Similar levels of physical activity as general people have been recommended in BCS [3]. This recommendation was reviewed by American College Sports Medicine experts in exercise for cancer who suggested the necessity to individualize the programs to cancer populations [4]. A recent meta-analysis concluded that exercise interventions should be multidimensional, including both exercise and behavioral interventions [3]. 

In fact, there is evidence that exercise and massage can be beneficial when tested as separate interventions for improving physical function in BCS [3]. A recent study has reported psychological and physical improvements after the application of a multimodal physical therapy program including in patients with different types of cancer [5]. Although conventional exercise programs [3] and alternative medicine approachs [6] applied on BCS with cancer-related fatigue have been previously studied, the application of core stability exercises (CSEs) as the main component of the program has not yet been investigated. 

CSEs are defined as exercises developing the ability to control the position and motion of the trunk during end-range segment in integrated kinetic chain activities [7]. It is known that BCS exhibit reduction in muscle strength associated with cancer-related symptoms [8], which could be improved with an exercise program including CSEs. 

Finally, disturbances of mood state have been reported as a frequent symptom in BCS [9]. Massage, which has been shown to be effective as a psychological resource [10, 11] and a recovery method after exercise [12] could be a main component of recovery process. Therefore, the aim of the current randomized controlled trial was to investigate the effectiveness of an 8-week physical therapy program focused on CSEs and recovery massage in physical (muscle strength) and psychological (mood state) outcomes in BCS. 

## 2. Methods

### 2.1. Subjects

Participants were recruited from the Breast Oncology Unit of Hospital Virgen de las Nieves, Granada, Spain from December 2008 to June 2010. The patients were approached and enrolled by physicians and nurses from two treatment departments. Participants were eligible if they (1) had a diagnosis of breast cancer (stage I–IIIA), (2) were 25–65 years, (3) finished coadjuvant treatment except hormone therapy, (4) not do have active cancer, and (5) present 4 or 5 of the following physical findings, judged by the oncologist who referred the patient: neck or shoulder pain, reduced range of motion in neck-shoulder region, reduced physical capacity, psychological problems, increased fatigue, sleep disturbances, or any problem in coping with physical and psychosocial functioning. They were excluded if they were receiving chemotherapy or radiotherapy treatment at the time of the study or they had chronic or orthopedic diseases which do not permit following the physical program. 

Potential participants were contacted by phone by 2 oncologists of the hospital. Those interested were cited for an appointment, received a complete explanation of the protocol and signed the consent form. The ethical approval for the study was granted by the Ethics Committee of the Hospital Virgen de las Nieves (no. 0890418, Granada, Spain). After inclusion, participants were scheduled for a medical visit including a history, physical examination, and a medical questionnaire. This visit had the goal of discovering conditions which justified any medical exclusion. 

### 2.2. Design, Randomization, and Allocation

A randomized controlled clinical trial was conducted. Eligible participants, after providing written informed consent, were randomly assigned into 2 groups: multimodal exercise group or a control group who received the usual care treatment for breast cancer. For ethical implications, those participants allocated to the control group, who finished the period of 6 months for the current study, were invited to be included into a new multimodal program or received an intervention by multimedia electronic document including exercises of all therapeutic sessions. We allocated patients to a multimodal program or control group in 4 randomization cycles, using computer-generated numbers. The sequence was entered into numbered opaque envelopes by an external member and they were opened after completion of the baseline assessment. 

### 2.3. Treatment: Multimodal Program

Multimodal program consisted of 24 hours of individual physical training and 12 hours of recovery procedures, conducted 3 times/week for 90 min each (Table 1). The intensity of the aerobic training was conducted following ACSM and AHA recommendations [13]. 

Physical training was followed by 30–40 min of low intensity interventions for improving recovery after exercise. This period included stretching of the muscles used during exercise and massage (myofascial release techniques) which has the ability to improve recovery after exercise [12]. 

After finish the 8 weeking supervised multimodal program, participants received an instructional DVD with the same exercise program which included aerobic exercise progression, resistance exercise, neck-shoulder mobility exercises, self-massage, and some relaxation techniques. The DVD included safety precautions related to exercise and health advice related to maintain and promote healthy lifestyle. 

### 2.4. Control Condition

Participants followed usual care recommended by the oncologist in relation with healthy lifestyle. A followup of the physical activity during control period was used to control possible bias detected in previous studies on exercise in BCS [3]. For that purpose, we used the Spanish version of Minnesota Leisure Time Physical Activity Questionnaire [14]. 

### 2.5. Data Analysis—Outcomes

The primary outcome was fatigue assessed using the fatigue subscale of Profile of Mood State (POMS) questionnaire. The POMS questionnaire (Spanish version) consists of 63 items on mood state. Scores (on a 5-point scale from 0 to 4) are grouped into six subscales: tension-anxiety, depression-dejection, anger-hostility, vigor, fatigue, and confusion. Subscale scores were converted into *T*-scores for the analysis, and the overall mood disturbance was also calculated. The reliability of the Spanish version of the POMS has been found to be high (Cronbach's *α* ranging 0.76–0.91) [15]. Assessors, participants, and therapists were blinded to the POMS scores during all the trial.

Secondary outcome measures included the following physical tests.

**(1) Trunk Curl Static Endurance Test**

This test requires a wedged piece of wood to support the patient at a fixed angle of 60°. The patients maintain both knees and hips flexed at 90°, the arms are folded across the chest and toes are anchored by the tester. The wood is pulled back 10 cm and the subject holds the isometric posture as long as possible. This test has proved to be reliable with coefficients of >0.97 for repeated tests [16].

**(2) Multiple Sit-to-Stand Test**

Participants were asked, while sitting at the front of a chair, to rise until they reached full knee extension and sit back 10 times as fast as they can. This test was used to assess general lower-extremity endurance [17]. This test has been showed reliable in similar age population [18].

All outcomes were completed before the program (pre-), immediately after the 8-week intervention (post-), and 6 months after discharge (followup). 

Based on a previous pilot study the sample size was calculated on an 80% power to detect a mean difference of 5 points, with a standard deviation of 4 (7%), on the POMS fatigue subscale, using a type 1 error (*α*) of 5%, and a type 2 error (*β*) of 20%. This power calculation resulted in 35 patients on each group. To accommodate expected dropouts before study completion, a total of 78 participants were included. 

### 2.6. Statistics

 Statistical analysis was performed using SPSS statistical software, version 19.0, and it was conducted according to intention to treat analysis principle. We used *t*-tests and Chi-square tests to examine differences in baseline sociodemographic and medical features between included and excluded patients, as well as between participants who completed the study and those who dropped out. A one-way ANOVA was used to compare both groups of BCS with healthy women from Hospital Virgen de las Nieves influence area (*n* = 43, age: 47 ± 12 years). 

The main analysis examined whether differences in outcomes (mean differences) among baseline, 8 weeks, and 6 months of followup existed between the groups. A 2 × 3 repeated-measure ANCOVA with intervention (experimental and control) as between-subjects variable, time (pre-, post-, and 6 months) as within-subjects variable, and age, status, educational level, and clinical features as covariates was used to examine the effects of the intervention on the main outcome. 

Intergroup effect sizes were calculated (*Cohen d*). An effect size <0.2 reflects a negligible difference, between ≥0.2 and ≤0.5 a small difference, between ≥0.5 and ≤0.8 a moderate difference, and ≥0.8 a large differences. The Pearson correlation test (*r*) was used to analyze the association between changes in mood state (mean differences) and in strength in the multimodal exercise group. A *P* < 0.05 was considered statistically significant.

## 3. Results

**Figure 1:**
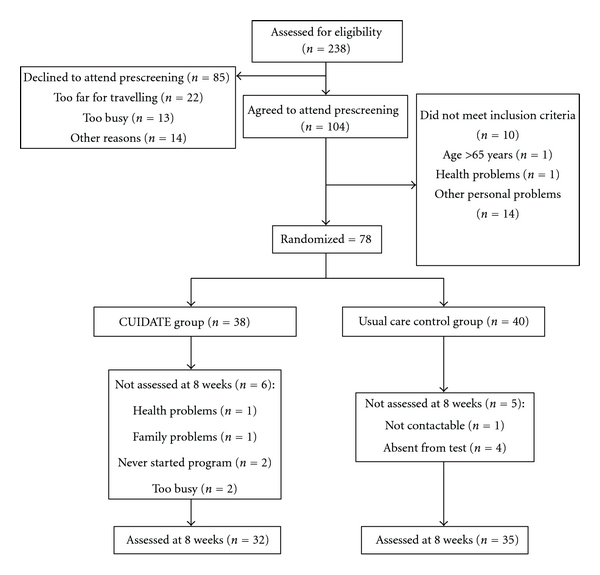
Flow diagram of subject recruitment and retention throughout the course of the study.

During the study period (from March 2009 to June 2010), 104 patients with cancer were agreed to attend prescreening (Figure 1). No differences in sociodemographic and medical features between the 78 patients (75%) included and the 26 patients (25%) who were excluded or declined to participate were found (Table 2). Participants who completed the study did not show differences in mood at baseline as compared to those who dropped out. The ANOVA revealed that patients in both groups had significantly disturbances of mood state in all subscales of the POMS as compared to healthy women (Table 3).

Patients who finished cancer treatment within the first 6 months before begining the multimodal exercise program completed 79.6% of the 24 physical therapy sessions (mean ± SD: 19 ± 5) whereas patients incorporated >6 months after finishing cancer treatment completed 87.4% of the 24 sessions (mean: 21 ± 6). No adverse effect was reported during the study. 

The ANCOVA found significant group × time interaction for the main outcome of the study, fatigue (*F* = 4.506; *P* = 0.015): the multimodal exercise group experienced a greater decrease of fatigue than the control group (Table 4). Intergroup effect sizes were moderate at postintervention (*d*: 0.52, 95% CI 0.14–0.81) and small at 6-month followup (*d*: 0.38, 95% CI 0.05–0.66).

Additionally, significant group × time interactions for the remaining domains of the POMS were also found: tension-anxiety (*F* = 5.918, *P* = 0.005); depression-dejection (*F* = 5.214, *P* = 0.01); anger-hostility (*F* = 5.082, *P* = 0.010); vigor (*F* = 6.090, *P* = 0.004), and also for total mood disturbance (*F* = 3.512, *P* = 0.037): the multimodal exercise group experienced a greater decrease of tension-anxiety, depression-dejection, or anger-hostility and a greater increase of vigor compared to the control group (Table 4). Intergroup effect sizes were large for both tension-anxiety (*d*: 1.05, 95% CI 0.54–1.55) and depression-dejection (*d*: 0.80, 95% CI 0.29–1.30) domains, and small for total mood disturbance (*d*: 0.40, 95% CI 0.16–0.65), anger-hostility (*d*: 0.40, 95% CI 0.16–0.63), and vigor (*d*: 0.35, 95% CI 0.18–0.67) domains after treatment. Intergroup effect sizes after 6-month followup were moderate for tension-anxiety (*d*: 0.76, 95% CI 0.20–1.31) and depression-dejection (*d*: 0.74, 95% CI 0.25–1.35), and small for anger-hostility (*d*: 0.39, 95% CI 0.12–0.67), vigor (*d*: 0.41 95% CI 0.16–0.69) and total mood disturbance (*d*: 0.32 95% CI 0.05–0.60). No group × time interaction for confusion was found (*F* = 0.831; *P* = 0.442). 

**Figure 2:**
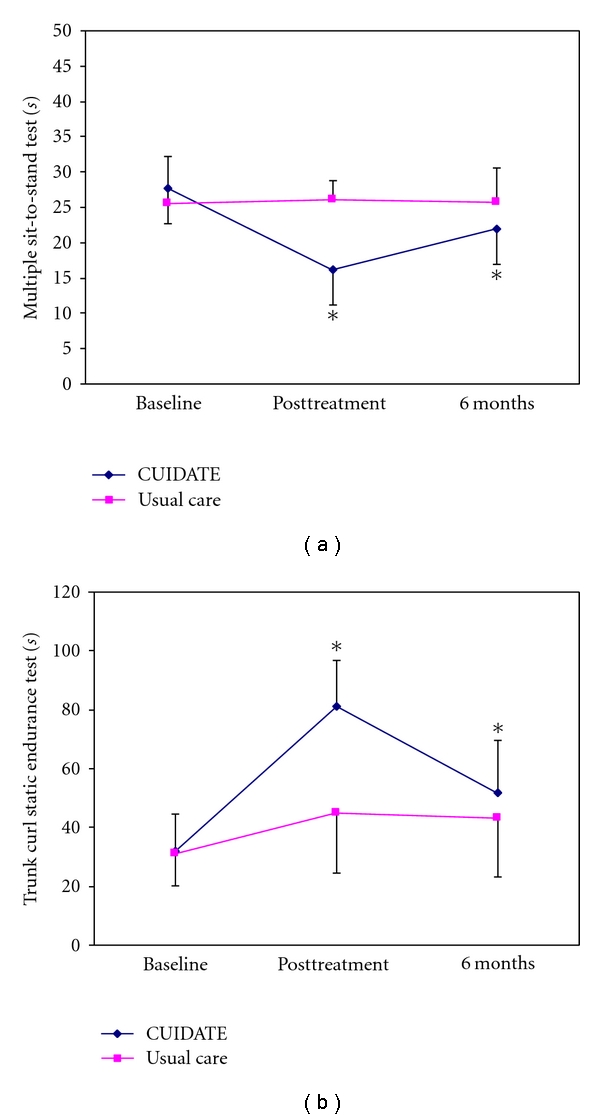
Multiple sit-to-stand test (*s*) and trunk curl static endurance test (*s*) changes after postintervention and 6 months followup, *significant changes respect baseline (*P* < 0.05).

A significant group × time interaction for multiple sit-to-stand test (*F* = 11.315; *P* < 0.001) and trunk curl static endurance test (*F* = 6.916; *P* = 0.002) was also found (Figure 2). Intergroup effect sizes were large for multiple sit-to-stand test (*d*: 0.96, 95% CI 0.71–1.20) and trunk curl static endurance test (*d*: 0.89, 95% CI 0.71–1.19) at postintervention, but moderate (multiple sit-to-stand test, 0.50 95% CI 0.27–0.90) and small (trunk curl static endurance test, 0.21 95% CI 0.20–0.47) at 6 month followup. 

A significant negative association (*r* = −0.352; *P* = 0.046) between changes in the total mood state and in the trunk curl static endurance test was found: the greater the decrease in mood, the higher the increase in muscle strength. 

## 4. Discussion

The current study found that an 8-week supervised multimodal program induced physical and psychological improvements in BCS. We noted a greater decrease on fatigue as compared to usual breast cancer care. The effects over fatigue were maintained at 6 months after discharge using DVD support. We also observed significant effects on other aspects of mood and physical capacity. 

 The effect size of the improvement in fatigue (0.52) suggests a medium clinically important change. Our results are relatively better from the findings of a recent meta-analysis which indicates that the magnitude of the effects from exercise interventions on CRS is small (effect size 0.31, 95% CI 0.22–0.40) [3]. Our study used similar length of treatment (8 weeks) than previous studies investigating exercise in CRF [3, 19, 20], but we extended postural control by including CSEs and combined movement on extremities which could explain our results. The results of the current study also showed that BCS within the first year after treatment exhibit more disturbances of mood state and fatigue than healthy women. At postintervention, mood disturbance improved in BCS within the multimodal program, reaching similar values to healthy women. On the contrary, BCS included in the control group continued exhibiting altered mood state as compared to healthy women. 

The POMS has been previously used to assess disturbance of mood state in oncology exercises studies [3]. Current results on mood state confirm the results from a previous pilot study using a similar exercise approach [21], since we found moderate-large effect sizes on several aspects of mood after the application of the multimodal program. Multimodal programs can help to BCS for coping with their cancer-related symptoms. Previous studies have suggested the necessity to apply interventions to better assist BCS for managing cancer related fatigue [19]. The multimodal program had a higher ratio of supervision with 2–4 therapists for 6–8 patients (ratio therapist/patient: 1/3–4). Only 60% of the exercise programs applied to reduce cancer related fatigue had employed therapist supervision [3], and the higher ratio therapist/patient of the multimodal program can promote social and environmental support, and satisfaction to the patients, both aspects which improve the mood state of BCS [22].

We also found significant and clinical improvement in muscle strength, which is consistent with recent studies on exercise [19, 20]. Current exercise guidelines for cancer apply minimal mention to muscle strength in BCS [23]. Our results suggest the necessity of including strength exercises in physical therapy programs for BCS. This may be related to the fact that cancer treatment, particularly chemotherapy, promotes disruption in muscle metabolism (i.e., adenosine triphosphate dysregulation, cytokine dysregulation, deprivation of satellite cells) wasting which may impair the maintenance of muscle mass [24]. CSEs were a major component of our program. Effectiveness of CSEs has been associated with modification of plasma levels of IL-6 and TNF-*α* by contraction of different muscles [25]. Interestingly, the current multimodal program produced large effect sizes in core-related muscles (trunk curl static endurance test) and also in nonrelated core muscles (leg muscles). These results may be explained because one of the principles of CSE is their ability to proximal muscle activation, providing interactive moments that would allow efficient distal muscle function [6]. Therefore, CSEs employed in our study may be also used for improving function of distal musculature through proximal (core-related) muscles.

One interesting result of our study was the relationship between the decrease in mood disturbance and the increase in strength of abdominal muscles. Cancer related fatigue constitutes a complex process involving both physical and psychosocial aspects [26]. Cancer patients who engage in negative beliefs about their cancer related symptoms (i.e., catastrophizing, fear of recurrence) are more likely to experience more intense symptoms [27]. It is possible that treatment programs combining preferred women's exercises [24] and recovery massage following an integrative oncology approach have a relevant role in mood improvement associated to increased functional state, as reflected in an increase of strength. 

One of the most important results of this trial is the maintained effects in mood and strength, although slightly reduced, after 6-month followup using a DVD support. This kind of strategy based on multimedia supporting promote exercise in BCS had shown good results in previous studies [28]. A mixed intervention, including an initial supervised phase focussed on proper learning of the exercise program, promotes high improvements in BCS. Nevertheless, after the program, DVD support is needed for maintaining the improvements during the treatment. Future studies investigating effects of supervised programs with a follow-up period based on telerehabilitation are needed. 

Strengths of the current trial include supervised and structured exercise program, multimodal cancer approach, use of validated objective measurements and a validated questionnaire, and intention-to-treat analyses; however, we should recognize that the control group was allowed to freely increase physical activity during the study. The possible bias [3] associated to this weakness was controlled since our control group did not show significant increases in physical activity during the study. 

## 5. Conclusions

In conclusion, an 8-week multimodal physical therapy program using CSE and massage recovery was clinically effective for improving physical (muscle strength) and psychological (mood state and fatigue) aspects in BCS as compared to usual treatment care. 

##  Funding

The authors certify that no party having a direct interest in the results of the research supporting this article has or will confer a benefit on us or on any organization with which we are associated with and, if applicable, the authors certify that all financial and material support for this paper (e.g., NIH or NHS grants) and work are clearly identified in the Acknowledgments section of the paper.

## Figures and Tables

**Table 1:** Description of the CUIDATE (intervention) program.

CUIDATE program		
Week 1–4		

Material	Small soft ball, mats, and fit-ball	

Endurance program	Unspecific work during sessions	

Exercise Program	Content	Dosage and progression

	(1) Half squat with arm movement	Week 1: Learning proposal. Assessment maximum load Week 2-3: 75% maximum load Increase 5% per week Continue progression between exercises: 2 sets/30 sec pause Week 4: 75% maximum load. Increase number series (3 sets) Medium velocity execution exercises Increase range of joint motion
	(2) Standing rows with leg semiflexion maintained	
	(3) Wall push-ups	
	(4) Abdominal with lower limb movement	
	(5) All tours with hip and knee movement	
	(6) Abdominal with adductor isometric contraction and armmovement	
	(7) Standing hip circumduction	
	(8) Supine on fit-ball with arm movements	
	(9) Superman on fit-ball	
	(10) Oblique partial sit-up	

Week 5–8		

Materials	Fit-ball, elastic band, mats, and small soft ball	

Endurance program	10–25 min of fast working with arms movement two days per week	

Exercise Program	Content	Dosage and progression

	(1) Chest press on fit-ball with elastic band	Week 5: 10–12 repetitions × 2 sets Week 6: 12–15 repetitions × 2 sets Week 7: 10–12 repetitions × 3 sets Week 8: 10–12 repetitions × 2 sets Increase resistance with elastic band and positions that require more body control
	(2) Squat with elastic band	
	(3) Seated rows on fit-ball with elastic band	
	(4) Isometric abdominal sitting on fit-ball with arm and leg movement	
	(5) Biceps curl on fit-ball with elastic band	
	(6) Biceps curl with elastic band and leg semiflexion maintained	
	(7) Leg curl with fit-ball	
	(8) Sit-up with lower limb movement	

**Table 2:** Patient's characteristics and comparisons between both breast cancer survivor groups.

Variable	Control Group (*n* = 35)	CUIDATE program (*n* = 32)	*P* value
Age (y), mean (SD)	48 (9)	49 (9)	0.415
Time after treatment, *n* (%)			
<12 months	29 (82.9)	22 (68.8)	0.176
>12 months	6 (17.1)	10 (31.3)	
Civil status, *n* (%)			
Married	21 (60.0)	20 (62.5)	0.718
Unmarried	8 (22.9)	5 (15.6)	
Divorced	6 (17.1)	7 (21.9)	
Educational level, *n* (%)			
Low	13 (37.1)	11 (34.4)	0.481
Medium	6 (17.1)	8 (25.0)	
University level	16 (45.7)	13 (40.6)	
Employment status, *n* (%)			
Home employed	8 (22.9)	7 (21.9)	0.586
Employed	14 (40.0)	10 (31.3)	
Un employed	13 (37.1)	15 (46.9)	
Tumor stage, *n* (%)			
I	12 (34.3)	4 (12.5)	0.145
II	16 (45.7)	23 (71.9)	
IIIA	7 (20.0)	5 (15.6)	
Type of surgery, *n* (%)			
Tumorectomy	21 (60.0)	21 (65.6)	0.596
Mastectomy	14 (40.0)	11 (34.4)	
Type of treatment *n* (%)			
Radiation	1 (2.9)	1 (3.1)	0.991
Chemotherapy	3 (8.6)	3 (9.4)	
Radiation + chemotherapy	31 (88.6)	28 (87.5)	
Menopause, *n* (%)			
Yes	20 (57.1)	24 (75.0)	0.197
Not	15 (42.9)	8 (25.0)	
Physical activity (METS/h*day)	7.94 (3.37)	8.63 (3.85)	0.364

*P values for comparisons among group based on Chi-square and analysis of variance tests.

**Table 3:** Comparison of Profile of Mood State (POMS) data among healthy reference women and breast cancer survivors at baseline.

POMS	Healthy women (*n* = 43)	CUIDATE program (*n* = 32)	CONTROL group (*n* = 35)	*P* CUIDATE versus control
Tension-anxiety^a^	37.93 ± 8.71	49.00 ± 10.44	50.14 ± 10.18	0.65
Depression-dejection^a^	42.56 ± 7.14	52.39 ± 12.14	52.42 ± 11.01	0.99
Anger-hostility^a^	46.66 ± 6.89	55.17 ± 11.99	57.03 ± 14.12	0.53
Vigor^a^	57.43 ± 6.61	48.17 ± 7.08	49.19 ± 6.47	0.52
Fatigue^a^	39.90 ± 5.61	51.48 ± 10.85	54.19 ± 10.09	0.24
Confusion^a^	32.86 ± 4.53	42.35 ± 9.68	44.30 ± 9.70	0.53
Disturbance^a^	−14223 ± 2743	−19942.85 ± 5901.69	−20845.15 ± 5299.82	0.61

^**a**^ *P* < 0.001 for ANOVA analysis among breast cancer survivors at baseline and healthy women.

**Table 4:** Preintervention, postintervention, and change scores for mean values of POMS.

Group	CUIDATE program	Control	Between-group differences
Tension-anxiety			

Preintervention	49.00 ± 10.44	50.14 ± 10.18	
Postintervention	39.33 ± 8.08	49.80 ± 10.32	
6 months followup	43.53 ± 9.62	51.12 ± 11.08	
Within group change scores			
Pre-post intervention	−9.66 (−13.45; −5.83)	−0.34 (−2.95; 2.26)	−9.32 (−13.79; −4.85)*
Pre intervention–6 months follow up	−5.89 (−2.53; −9.54)	−0.28 (−2.76; 6.26)	−6.17 (−1.71; −10.63)

Depression-dejection			

Preintervention	52.39 ± 12.14	52.42 ± 11.01	
Postintervention	47.15 ± 9.34	52.40 ± 10.91	
6 months followup	48.17 ± 8.94	55.30 ± 12.12	
Within group change scores			
Pre-post intervention	−7.36 (−11.15; −3.57)	−0.02 (−2.84; 2.79)	−7.33 (−11.93; −2.73)*
Pre intervention–6 months follow up	−4.22 (−8.62; −0.87)	2.88 (0.73; 6.50)	−7.00 (−12.64; −0.77)

Anger-hostility			

Preintervention	55.17 ± 11.99	57.03 ± 14.12	
Postintervention	46.82 ± 9.14	58.34 ± 11.65	
6 months followup	49.25 ± 8.07	58.76 ± 13.17	
Within group change scores			
Pre-post intervention	−7.87 (−12.16; −3.59)	1.31 (−2.05; 4.04)	−9.19 (−14.20; −3.65)*
Pre intervention–6 months follow up	−5.92 (−10.13; −1.72)	1.73 (−1.59; 5.06)	−7.65 (−12.95; −2.36)

Vigor			

Preintervention	48.17 ± 7.08	49.19 ± 6.47	
Postintervention	53.46 ± 8.02	49.29 ± 7.31	
6 months followup	53.17 ± 8.41	48.00 ± 6.98	
Within group change scores			
Pre-post intervention	5.29 (3.40; 8.29)	0.17 (−2.57; 2.22)	5.12 (2.65; 9.38)*
Pre intervention–6 months follow up	5.00 (2.16; 7.83)	−1.19 (−3.94; 1.56)	6.19 (2.30; 10.06)

Fatigue			

Preintervention	51.58 ± 10.85	54.19 ± 10.09	
Postintervention	43.93 ± 8.58	52.26 ± 10.09	
6 months followup	45.12 ± 10.31	53.34 ± 9.36	
Within group change scores			
Pre-post intervention	−8.03 (−11.19; −4.86)	−1.93 (−5.06; 0.20)	−6.10 (−9.12; −1.07)*
Pre intervention–6 months followup	−6.45 (−9.50; −3.39)	−0.84 (−3.44; −1.74)	−5.61 (−8.56; −0.35)

Confusion			

Preintervention	42.35 ± 9.68	44.30 ± 9.70	
Postintervention	37.67 ± 7.08	42.90 ± 8.82	
6 months followup	39.85 ± 9.48	43.70 ± 9.44	
Within group change scores			
Pre-post intervention	−4.68 (−7.71; −1.55)	−1.40 (−4.55; 1.11)	−3.28 (−7.05; 1.22)
Pre intervention–6 months follow up	−2.50 (−5.36; 0.36)	−0.60 (−4.39; 3.19)	−2.91 (−6.42; 2.62)

Total disturbance mood			

Preintervention	−19942.85 ± 5901.69	−20845.15 ± 5299.82	
Postintervention	−16000.00 ± 3532.28	−20353.84 ± 5888.03	
6 months followup	−17257.14 ± 4528.05	−20884.61 ± 6171.78	
Within group change scores			
Pre-post intervention	3442.85 (1623.71; 5353.11)	491.31 (−905.90; 1608.76)	2951.54 (754.29; 5124.67)*
Pre intervention–6 months follow up	2685.71 (986.08; 4835.34)	38.46 (−1553.29; 1630.21)	2647.25 (454.29; 4854.29)

*Significant group × time interaction (Repeated ANOVA test, *P* < 0.05).
